# Reference parameters for normality and associated factors to hard palate during mixed dentition phase

**DOI:** 10.1590/2317-1782/20212020291

**Published:** 2021-10-25

**Authors:** Luana Cristina Berwig, Mariana Marquezan, Jovana de Moura Milanesi, Jessica Klöckner Knorst, Thiago Machado Ardenghi, Ana Maria Toniolo da Silva

**Affiliations:** 1 Serviço de Fonoaudiologia, Hospital de Clínicas de Porto Alegre - HCPA - Porto Alegre (RS), Brasil.; 2 Departamento de Estomatologia, Faculdade de Odontologia, Universidade Federal de Santa Maria – UFSM - Santa Maria (RS), Brasil.; 3 Departamento de Fonoaudiologia, Curso de Fonoaudiologia, Universidade Federal de Santa Maria – UFSM - Santa Maria (RS), Brasil.

**Keywords:** Hard Palate, Mixed Dentition, Orthodontics, Speech-Language Pathology, Observational Study, Palato Duro, Dentição Mista, Ortodontia, Fonoaudiologia, Estudo Observacional

## Abstract

**Purpose:**

To evaluate the association among dimensions of the hard palate according to the sexes, skin color, and periods of the mixed dentition and present reference parameters of normality for this stage of development.

**Methods:**

This cross-sectional study evaluated a representative sample of children between the ages of 7 and 13 years in Santa Maria, southern Brazil. The outcomes of the study were the dimensions of the palate: width measurements and depth. Sociodemographic characteristics and related oral measures were also assessed. Adjusted linear regression model were used to evaluate the effect of the predictor’s variables on the dimensions of the hard palate in millimeters. The reference standards for the hard palate normality were presented in mean, standard deviation, and 95% confidence interval.

**Results:**

A total of 569 children were evaluated. The hard palate dimensions were larger in the male sex and smaller in the first transitional period of mixed dentition. Skin color had an influence on the hard palate width at the level of the premolars, and the width measurements were smaller in white individuals. Posterior cross bite, Angle Class II and III malocclusions and non-nutritional sucking habits caused reduction in the hard palate width measurements.

**Conclusion:**

Different dimensions of the hard palate are influenced by demographic variables such as sex, skin color, and mixed dentition period. Children of the female sex, white-skinned and in the first transition period of mixed dentition had smaller dimensions of the hard palate. Establishing normality reference standards in measurements of the palates guides the clinical practice.

## RESUMO

**Objetivo:**

Avaliar a associação entre as dimensões do palato duro de acordo com os sexos, cor da pele e períodos da dentição mista e apresentar parâmetros de normalidade de referência para esta fase de desenvolvimento.

**Método:**

Este estudo transversal avaliou uma amostra representativa de crianças com idade entre 7 e 13 anos em Santa Maria, sul do Brasil. Os desfechos do estudo foram as dimensões do palato: medidas de largura e profundidade. Características sociodemográficas e medidas orais relacionadas também foram avaliadas. Modelos de regressão linear ajustados foram utilizados para avaliar o efeito das variáveis preditoras sobre as dimensões do palato duro em milímetros. Os padrões de referência para a normalidade do palato duro foram apresentados em média, desvio padrão e intervalo de confiança de 95%.

**Resultados:**

Foram avaliadas 569 crianças. As dimensões do palato duro foram maiores no sexo masculino e menores no primeiro período de transição da dentição mista. A cor da pele influenciou a largura do palato duro ao nível dos pré-molares, sendo as medidas de largura menores nos brancos. Mordida cruzada posterior, más oclusões de Classe II e III de Angle e hábitos de sucção não nutricionais causaram redução nas medidas da largura do palato duro.

**Conclusão:**

As diferentes dimensões do palato duro são influenciadas por variáveis demográficas como o sexo, cor da pele e período de dentição mista. Crianças do sexo feminino, de cor de pele branca e no primeiro período de transição de dentição mista apresentaram menores dimensões do palato duro. O estabelecimento de padrões de referência de normalidade nas medidas do palato orienta a prática clínica.

## INTRODUCTION

The hard palate is one of the structural components of the oral sensorimotor system. Its anatomical and dimensional integrity is important for adequate processing of the functions of sucking, swallowing, chewing and phonoarticulation^(1)^. The morphological constitution of this structure is also closely related to the alveolar topography and dental occlusion^(2-4)^. In view of this, examination of the hard palate is imperative for establishing the functional and occlusal therapeutic plan.

In clinical practice, the exam related to the size of the hard palate frequently raises doubts, bearing in mind the subjectivity of the evaluation criteria^(1)^. In the literature, there are different methods of quantitative evaluation: measurements made with a pachymeter on plaster models^(5)^, in the mouth or model, using a three-dimensional compass^(6-8)^, in digitized plaster models with the use of software^(9)^ and on plaster models with the use of a digital pantograph^(10)^. In spite of this diversity of instruments, a lack of studies with representative samples has been verified, which present standard reference measurements of the hard palate in children.

In this sense, this investigation was motivated by the need for reference standards for quantitative analysis of the hard palate, bearing in mind that no studies conducted with representative samples in south Brazil were found. Establishing normality reference standards for measurements of the palates extremely important, as it guides the professional when making decisions in clinical practice. In this sense, since abnormalities related to the measurements of the hard palate can be related to the occurrence of malocclusions^(11)^, the correct intervention can improve the well-being and quality of life of the patient, especially throughout adolescence^(12,13)^. In addition, recognizing the factors associated with abnormal patterns allows specific interventions in these groups.

The aims of this study were as follows: present reference parameters of normality for quantitative analysis of the hard palate in children in the mixed dentition stage; and verify whether there are differences between the sexes, skin color, and periods of mixed dentition. We hypothesized that the measurements of the hard palate were influenced by demographic and clinical variables in the sample.

## METHODS

### Study design and sample

This is a cross-sectional study performed with a random sample of children between 7 and 13 years old (mixed dentition stage) from Santa Maria, Southern Brazil. In the year 2015, the municipality had an estimated population of 261,031 inhabitants, of whom 30,216 (11.57%) were enrolled in the primary education system^(14)^. Of these children, about 10,569 children were enrolled in the 26 primary schools of the state network. The random two-stage cluster sampling procedure was adopted, so that nine of these schools were randomly selected, according to the different administrative regions and size of the school. Therefore, the schools were considered the primary sample unit, and the children, the secondary sample unit.

The sample size was calculated considering the difference of means of the palatal depth of 21.7 (SD 1.5) and 20.7 (SD 1.3) in males and females, respectively^(15)^. For this approach, a significance level of 5% and power of 80% were used. Considering a design effect of 1.2 and adding 20% for possible losses, the minimum sample required was 544 participants. Included in the study were all the children enrolled in the nine selected schools who were in the mixed dentition stage and with the maxillary first molars erupted. Excluded from the study were those who presented evident signs of syndromes and/or cognitive limitations, and/or presented a history of orthodontic treatment.

### Data collection

Data collection was conducted throughout 2015 including structured interviews and clinical examinations of the children. The interviewers underwent training involving theoretical discussions and practical training. All children were examined at the schools by four previously trained and calibrated dentists and one Speech-language pathologist. The calibration and data collection process were carried out according to international criteria standardized by the World Health Organization for oral health^(16)^.

### Quantitative evaluation of the hard palate (outcome variables)

The transverse dimensions (width) of the hard palate were obtained directly in the oral cavity, with the use of a digital pachymeter (Digimess^®^, Brazil), with a resolution of 0.01 mm and precision of 0.03 mm. The vertical dimension (depth) was measured with a Korkhaus (Dentaurum®, Germany) three-dimensional compass, which has been used in other previous studies^(6,7)^. This evaluation was performed at the schools, with the children positioned in a reclining chair. The examiner made use of a headlamp for correct visualization of the points of reference.

The points of reference in the region of the canine teeth, premolars (or deciduous molars) and permanent molars were the most apical portion of the gingival margin^(17,18)^. The width measurements corresponded to the transverse distance in millimeters between the points of reference of the teeth considered. The hard palate depth measurement corresponded to the vertical measurement in millimeters obtained from the median palate raphe up to the region that united the reference points of the second premolar teeth or deciduous second molars. The measurements were not taken in situations in which there were absence of one or both reference teeth, or if there were caries lesions or trauma that would change the perimeter of the dental arch.

Quantitative evaluation of the hard palate was made by the same previously calibrated speech/language therapist to obtain all the measurements established. For this purpose, 30 children were re-evaluated after an interval of one week, to obtain the intra-evaluator reproducibility by means of the Intraclass Correlation Coefficient: (ICC): canine width (ICC = 0.95); first premolar width or deciduous first molar (ICC= 0.98); second premolar width or deciduous second molar (ICC = 0.96), first molar width (ICC = 0.90), second premolar depth or deciduous second molar (ICC = 0.90).

### Orthodontic evaluation

The children were examined at the schools by four previously trained and calibrated dentists (Kappa intra- and inter-examiner >0.70) for evaluation of all the oral health variables considered. These exams were performed to classify the period of dentition (deciduous, mixed or permanent); and the periods of mixed dentition (first transitional period, inter-transitional period and second transitional period). In addition, the exams were performed to evaluate occlusal changes that could have an effect on the measurements of the hard palate (outcomes): anterior open bite (present/absent), unilateral or bilateral posterior cross bite (present/ absent) and presence of Angle’s Class I, II and III malocclusions (present/absent).

### Speech-language evaluation

The children were evaluated by a single calibrated Speech-language pathologist (Kappa > 0,70) by means of application of the Protocol of Orofacial Myofunctional Evaluation with Scores^(19)^. According to this protocol, are attributed scores for breathing mode on a 4-point scale: (4) = “normal patter”, when the lips remained in occlusion without effort, mainly during situations of rest and mastication, with the tongue contained in the oral cavity); (3) = “mild alteration”, when the subject presented oronasal inspiration but was able to perform inspiration only through the nose without showing signs of fatigue and dyspnea, (2) = “moderate alteration” when the condition was similar to the previous one but the subject did not maintain a nasal pattern; and (1) = “severe alteration”, when the subject, while trying to perform nasal only inspiration, showed signs of fatigue and dyspnea and opened his mouth to inspire within a few seconds, a pattern observed both at rest and during mastication^(19)^. From this evaluation, the presence (score 1, 2 or 3) or absence (score 4) of light or severe oronasal respiration was verified.

### Covariables

By means of structured questionnaires, information was obtained related to the sociodemographic characteristics and included: age (years), sex (male or female) and skin color. Skin color was collected according to the criteria established by Brazilian Institute of Geography and Statistics (IBGE) into white, black, brown, yellow and indigenous people^(13)^ and dichotomized in white and Non-white (black, brown, yellow and indigenous). Behavior’s measurements were also evaluated and included non-nutritional sucking habits (pacifier and finger sucking). Non-nutritional sucking for a prolonged time was considered use of pacifier and/or finger sucking for three years or longer^(19)^.

### Data analysis

The SPSS 20.0 program was used to analyze the data. Initially, descriptive analysis of the sample profile was made of the variables selected for evaluating the effect on the hard palate measurements. By using the Analysis of Variance (ANOVA) and t-test the means of the hard palate dimensions obtained were compared between the sexes, periods of the mixed dentition, and skin color.

Adjusted linear regression was used to evaluate the effect of the predictor’s variables on the dimensions of the hard palate. Variables with P value < 0.20 in the unadjusted analysis were considered for the adjusted model. The results are presented as beta coefficient (β) and its respective p-value (p<0.05 were considered significant). For establishing the reference standards for the quantitative analysis of the hard palate, the mean, standard deviation and 95% confidence interval (95% CI) of the measurements were presented, excluding all the children who presented one or more variables that were shown to have effect on the hard palate measurements in the adjusted linear regression.

### Ethical aspects

The Research Ethics Committee of the Federal University of Santa Maria approved this research (Protocol No. 08105512.0000/5346). The parents and legal guardians of the participants signed the Term of Free and Informed Consent (TFIC). All the schools were provided with information about the study aims and procedures and agreed to participate by means of signing the Term of Institutional Authorization. A letter of invitation informing about the research, questionnaires, and two copies of the TFIC were sent to the potential participants’ homes.

## RESULTS

**Figure 1:**
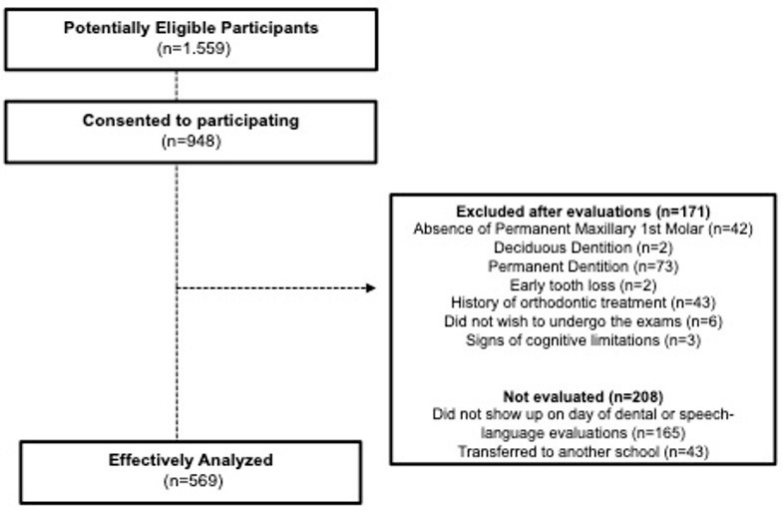
Flow diagram with the number of participants in each stage of the study

From 1,559 children invited to participate in the study, of these, 948 agreed and had the term signed by the person responsible for them (response rate of 60.8%). Of these, 171 were excluded after the orthodontic and/or Speech-Language evaluations, because they did not meet the criteria, and 208 were excluded because they had not undergone any of the evaluations or did not complete the two evaluations proposed in this study. Thus, 569 children were effectively included in the analysis of the data (Figure 1).

Table 1 shows the characteristics of the sample. Similar distribution was verified between the sexes, predominance of white children (76.4%), and that the majority of the children were in the second transitional period of mixed dentition (40.6%). The mean age was 9.66 (SD 1.55) and 9.74 (SD 1.60) years in females and males, respectively. As expected, the mean age increased progressively according to the periods of mixed dentition. The majority of the children presented no habit of sucking for three years or longer (64.8%). Relative to the clinical characteristics, higher prevalence was verified of the nasal breathing mode, Angle’s Class I malocclusion, and low prevalence of anterior open bite and posterior cross bite.

**Table 1:** Sample profile according to sociodemographic characteristics, non-nutritional sucking habits, respiratory mode, periods of mixed dentition and occlusal changes (n = 569)

**Variables**	**n (%)**	**Mean (SD)**
**Sex**		
Female	300 (52.7)	-
Male	269 (47.3)	-
**Skin color**		
White	435 (76.4)	-
Non-whites	122 (21.4)	-
**Periods of mixed dentition**		
1st transitional period	189 (33.2)	-
Intertransitional period	144 (25.3)	-
2nd transitional period	231 (40.6)	-
**Mean age according to sex**		
Female	-	9.66 (1.55)
Male	-	9.74 (1.60)
**Mean age in mixed dentition periods**		
1st transitional period	-	8.18 (0.88)
Intertransitional period	-	9.74 (1.18)
2nd transitional period	-	10.89 (1.10)
**Non-nutritional suction for prolonged period of time**		
No	369 (64.8)	-
Yes	195 (34.3)	-
**Respiratory mode**		
Nasal	357 (62.7)	-
Light and Severe Oronasal Breathing	212 (37.3)	-
**Anterior open bite**		
Absent	478 (84)	-
Present	41 (7.2)	-
**Uni or bilateral cross bites**		
Absent	500 (87.9)	-
Present	59 (10.4)	-
**Angle’s classification**		
Normal or Class I occlusion	478 (84.0)	-
Class II, 1st division	60 (10.5)	-
Class II, 2nd division	13 (2.3)	-
Class III	14 (2.5)	-

Values less than 569 are due to missing data; SD, standard deviation.

In Table 2 the means of the transverse and vertical dimensions of the hard palate were compared among the children of different sexes, skin color and period of mixed dentition. The means of the hard palate dimensions were found to be significantly higher in male sex (p<0.001). There was no difference in the dimensions of the hard palate between whites and non-whites. Relative to the period of mixed dentition, all the transverse dimensions were verified to be significantly smaller in the first transitional period when compared with the other periods (p<0.001). The depth at the level of the deciduous second molars or second premolars presented significant growth in values as the mixed dentition period advanced (p<0.001).

**Table 2:** Width and depth dimensions of the hard palate expressed as mean and standard deviations according to sex, skin color and period of mixed dentition

**Variables**	**Hard palate dimensions in millimeters (mm) / Mean (SD)**				
	**W.Ca**	**W.1PM**	**W.2PM**	**W.1M**	**Dep.2PM**
**Sex**					
Female	25.89 (2.28)	27.33 (2.17)	30.56 (2.40)	33.35 (2.27)	9.32 (1.55)
Male	26.76 (2.28)	28.45 (2.39)	31.88 (2.38)	34.74 (2.43)	9.66 (1.55)
p -value^(1)^	<0.001	<0.001	<0.001	<0.001	0.01
**Skin color**					
White	26.31 (2.34)	27.80 (2.32)	31.09 (2.45)	33.97 (2.42)	9.45 (1.50)
Non-whites	26.25 (2.31)	28.11 (2.43)	31.56 (2.54)	34.24 (2.54)	9.66 (1.76)
p -value^(1)^	0.81	0.24	0.07	0.27	0.20
**MD Period**					
1st T period	25.65 (2.23)^A^	27.42 (2.23^A,C^	30.36 (2.31)^A^	33.36 (2.46)^A^	8.86 (1.29)^A^
IT Period	26.91 (2.20)^B,C^	28.54 (2.27)^B^	31.45 (2.17)^B,C^	34.37 (2.04)^B,C^	9.50 (1.55)^B^
2nd T period	26.61 (2.37)^C,B^	27.82 (2.39)^C,A^	31.72 (2.63)^C,B^	34.33 (2.58)^C,B^	9.99 (1.59)^C^
p -value^(2)^	<0.001	<0.001	<0.001	<0.001	<0.001

1 Student’s t Test;

2 Analysis of Variance (ANOVA);

A,B,C Difference in the Tukey test between different superscript letters; SD, Standard Deviation; MD, mixed dentition; T, transitional; IT, intertransitional; W.Ca, Canine Width; W.1PM, lst premolar width or deciduous first molar; W.2PM, Second premolar width or deciduous second molar; W.1M, 1st molar width; Dep.2PM, second premolar depth, or deciduous second molar

Table 3 presents the results of the adjusted linear regression analysis performed to evaluate the effect of the predictors variables on the hard palate. There was evidence of the negative effect of the variables white skin color, habit of non-nutritional sucking for a prolonged period of time, posterior cross bite, and Angle’s Class II and Class III malocclusion on the transverse measurements of the hard palate (p<0.05). The variables of male sex and increase in age, presented positive effects in measures of the hard palate (p<0.05). In addition, the variables oronasal respiratory mode and increase in age had also a positive effect on the depth of the hard palate (p<0.05).

**Table 3:** Adjusted linear regression analysis to evaluate the factors associated with the hard palate measurements

**Outcomes**	**Predictors**	**β**	**p-value**	**R^2^**
W.Ca				17.2%
Pachymeter	Age	0.165	<0.001	
(mm)	Male sex	0.195	<0.001	
	SNN for 3 years or longer	-0.114	0.021	
	Posterior cross bite	-0.233	<0.001	
W.1PM				16.2%
Pachymeter	Male sex	0.230	<0.001	
(mm)	White race	0.104	0.022	
	Malocclusion Class II and III	-0.117	0.010	
	SNN for 3 years or longer	-0.146	0.003	
	Posterior cross bite	0.166	<0.001	
W.2PM				21.8%
Pachymeter	Age	0.223	<0.001	
(mm)	Male sex	0.247	<0.001	
	White race	-0.113	0.008	
	Malocclusion Class II and III	-0.114	0.007	
	Posterior cross bite	-0.211	<0.001	
W.1M				18.6%
Pachymeter	Age	0.176	<0.001	
(mm)	Male sex	0.264	<0.001	
	Malocclusion Class II and III	-0.124	0.003	
	Posterior cross bite	-0.211	<0.001	
Dep.2PM				18.3%
Compass	Age	0.346	<0.001	
(mm)	Oronasal breathing mode	0.225	<0.001	

β, beta coefficient; R2, coefficient of determination; W.Ca, Canine Width; W.1PM, lst premolar width or deciduous first molar; W.2PM, Second premolar width or deciduous second molar; W.1M, 1st molar width; Dep.2PM, second premolar depth, or deciduous second molar.; mm, millimeters

Reference parameters for quantitative analysis of the hard palate according to the periods of mixed dentition, sex, and skin color are presents in Table 4. The reference parameters of canine width for individuals of the female sex and white skin color in the 1st transition period were 26.3 (SD 1.9) [CI 22.5-30.0] millimeters. In relation to 1st molar width in the second transition period, the reference parameters were 35.5 (SD 2.2) [CI 31.1-39.8] and 37.0 (SD 2.7) [CI 31.7-42.2] millimeters for boys of white and no-white skin color, respectively. Children who presented variables that significantly influenced the on the width and depth measurements of the hard palate according to the adjusted analysis (non-nutritional sucking for a prolonged period of time, posterior crossbite, Angle’s Class II and Class III, oronasal respiratory mode) were excluded.

**Table 4:** Reference parameters for quantitative analysis of the hard palate, expressed in millimeters (mean, standard deviation and interval of confidence) according to the periods of mixed dentition, sex and skin color

	**Periods of mixed dentition**											
	**1St Transitional Period - 8.18 (SD 0.88) years of age**				**Intertransitional Period - 9.74 (SD 1.18) years of age**				**2nd Transitional Period - 10.89 (SD 1.10) years of age**			
	**Female Sex**		**Male Sex**		**Female Sex**		**Male Sex**		**Female Sex**		**Male Sex**	
**Parameters**	Skin color	Others	Skin color	Others	Skin color	Others	Skin color	Others	Skin color	Other races	Skin color	Other races
**(mm)**	White	Races	White	Races	White	Races	White	Races	White	(n=5)	White	(n=11)
	(n=34)	(n=8)	(n=26)	(n=3)	(n=21)	(n=4)	(n=20)	(n=5)	(n=38)		(n=22)	
	Mean (SD)	Mean (SD)	Mean (SD)	Mean (SD)	Mean (SD)	Mean (SD)	Mean (SD)	Mean (SD)	Mean (SD)	Mean (SD)	Mean (SD)	Mean (SD)
	[CI 95%]	[CI 95%]	[CI 95%]	[CI 95%]	[CI 95%]	[CI 95%]	[CI 95%]	[CI 95%]	[CI 95%]	[CI 95%]	[CI 95%]	[CI 95%]
**W.Ca**	26.3 (1.9)	26.4 (2.0)	26.4 (1.9)	26.2 (2.7)	26.6 (1.6)	26.2 (1.3)	27.7 (2.6)	28.4 (2.2)	27.0 (2.0)	-	27.4 (1.8)	27.4 (1.9)
**Pachymeter**	[22.5-30.0]	[22.5-30.3]	[22.6-30.1]	[20.9-31.4]	[23.4-29.7]	[23.6-28.7]	[22.6-32.7]	[24.0-32.7]	[23.0-30.9]		[23.8-30.9]	[23.6-31.1]
**W.1PM**	27.9 (1.9)	28.3 (2.0)	28.2 (2.2)	30.0 (2.9)	28.3 (1.7)	28.1 (1.1)	29.6 (2.4)	30.0 (2.2)	27.6 (1.8)	25.0 (1.7)	28.5 (2.2)	30.2 (2.4)
**Pachymeter**	[24.1-31.6]	[24.3-32.2]	[23.8-32.5]	[24.3-35.6]	[24.9-31.6]	[25.9-30.2]	[24.8-34.3]	[25.6-34.3]	[24.0-31.1]	[21.6-28.3]	[24.1-32.8]	[25.4-34.9]
**W.2PM**	30.8 (2.2)	30.5 (2.4)	31.2 (2.2)	31.9 (2.7)	31.1 (2.2)	31.2 (0.6)	32.5 (2.0)	32.1 (2.9)	31.6 (2.1)	31.9 (2.7)	32.6 (2.1)	34.0 (2.6)
**Pachymeter**	[26.5-35.1]	[25.8-35.2]	[26.8-35.5]	[26.6-37.1]	[26.7-35.4]	[30.0-32.3]	[28.5-36.4]	[26.4-37.7]	[27.4-35.7]	[26.6-37.1]	[28.4-36.7]	[28.9-39.0]
**W.1M**	33.8 (2.1)	33.0 (2.1)	34.1 (2.2)	35.3 (2.9)	34.0 (1.9)	34.2 (0.6)	35.0 (2.0)	35.0 (2.3)	34.2 (2.9)	34.0 (1.6)	35.5 (2.2)	37.0 (2.7)
**Pachymeter**	[29.7-37.9]	[28.8-37.1]	[29.7-38.4]	[29.6-40.9]	[30.2-37.7]	[33.0-35.3]	[31.0-38.9]	[30.4-39.5]	[28.5-39.8]	[30.8-37.1]	[31.1-39.8]	[31.7-42.2]
**Dep.2PM**	8.7 (0.9)	8.2 (2.4)	8.7 (1.0)	8.5 (2.1)	8.8 (1.5)	10.7 (1.9)	9.5 (1.4)	9.9 (1.9)	9.5 (1.4)	8.8 (1.0)	9.5 (1.5)	10.5 (1.8)
**Compass**	[6.9-10.4]	[3.4-12.9]	[6.7-10.6]	[4.3-12.6]	[5.8-11.7]	[6.9-14.4]	[6.7-12.2]	[6.1-13.6]	[6.7-12.2]	[6.8-10.7]	[6.5-12.4]	[6.9-14.0]

SD, Standard deviation, CI, confidence interval; W.Ca, Canine Width; W.1PM, lst premolar width or deciduous first molar; W.2PM, Second premolar width or deciduous second molar; W.1M, 1st molar width; Dep.2PM, second premolar depth, or deciduous second molar.; mm, millimeters

## DISCUSSION

This study aimed to present reference parameters of normality for quantitative analysis of the hard palate in children in the mixed dentition stage. Our findings showed that the hard palate dimensions were affected by sex, skin color, and period of mixed dentition, in agreement with the conceptual hypothesis. In addition, the presence of malocclusions and habits of non-nutritional sucking for a prolonged period of time caused a reduction in the hard palate width measurements, while the oronasal breathing mode causes an increase in depth. This investigation was motivated by the need for reference standards for quantitative analysis of the hard palate in representative samples in south Brazil, sustained the external validity of the results.

The mean age values verified in the three periods of mixed dentition were in agreement with the expected values^(20)^. The first transitional period occurred at around six to 8 years of age and was characterized by eruption of the permanent first molars and transition of the incisors. Whereas, the intertransitional period occurred around nine years of age, and lasted approximately one and a half years, in which vestibular and distal inclination of the incisor crowns was verified, frequently with the presence of diastemas. Finally, the second transitional period occurred at around ten to 12 years of age, and was characterized by eruption of the permanent canines, premolars and second molars^(20)^. Regarding to comparison of the hard palate dimensions in the periods of mixed dentition, influence of the development of dentition on the size of the hard palate could be suggested. All the transverse dimensions and depth of the hard palate measurements were verified to be significantly smaller in the children in the first transitional period, when compared with the other periods, according to previous studies^(21)^.

With regard to comparison of the hard palate measurements according to sex, all the dimensions of the hard palate were verified to be significantly larger in the male sex, corroborating the findings in the literature^(15,21-23)^. These findings can be explained by the fact that female individuals have smaller bony ridges and alveolar processes, in addition to having a weaker musculature, which has important effects on measurements of facial breadth and dental arch height and width^(15,24)^, and consequently, in the width of the hard palate.

Regarding skin color variable, were demonstrated that white skin color was found to have a negative effect on the measurements of width at the level of the first and second premolars; that is to say, white children presented a reduction in these measurements in comparison with non-white children, in agreement with a previous study^(25)^. A possible explanation for these results is differences in proportion and shape between some oral structures between whites and non-whites. The buccal cusps from the canine through the second premolar define a convex curve in blacks, while the canine and premolars are in an approximately straight line in whites. In this sense, whites had a more rounded and small arch, while blacks present a square and larger arch, in addition to a greater proportion of molar width^(25,26)^. In this sense, whites have a significantly smaller palatal index and arch length, and consequently, a reduction in measurements.

From the joint analysis of the variables that had an effect on the hard palate dimensions, the posterior crossbite was shown to be the clinical variable that had most influence on all the transverse dimensions of the palate, confirming the reduction in the width of the hard palate in children who had changed in this occlusal parameter^(27,28)^. The Angle Class II and III malocclusions also had a negative effect on the width measurements at the level of the premolars and molars, predisposing individuals towards narrowing of the hard palate^(29)^. The habits of non-nutritional sucking for three years or longer were also shown to have a negative effect on the most anterior region of the hard palate, corroborating the findings of previous studies^(19)^. In addition, the slight or severe oronasal respiratory mode was shown to have an influence on the depth of the hard palate, suggesting that children with this condition had a deeper hard palate than those with a nasal respiratory mode, which was in agreement with the literature at present^(6,7,9,19)^.

The reference parameters for qualitative analysis of the hard palate of children in the mixed dentition stage were reported considering sex and skin color, excluding those who presented one or more characteristics that also demonstrated an effect on the hard palate dimensions in the adjusted linear regression analysis. As regards the data related to the reference parameters, a limitation was verified in relation to the distribution of the number of non-white children in the 12 strata formed. It was also possible to find that the hard palate dimensions did not present a linear increase in the periods of mixed dentition, as has been observed in other studies conducted with children in the same age range^(8,22)^. This finding may be related to the dynamism of this period of development of occlusion, in which diverse dental changes are observed.

This study has some limitations. From the analyses performed, were demonstrated the effect of some variables on the dimensions of the hard palate. However, we point out that other variables, not considered, such as the facial growth pattern and muscular constitution, for example, may also have an influence on the hard palate dimensions. In addition, the majority of children evaluated were of the white skin color. However, this values are in accordance with a previous study conducted with a representative sample of schoolchildren in the city of Santa Maria^(30)^. Notwithstanding, no Brazilian studies with representative samples relative to this topic were found, indicating the need for conducting new researches in other populations so that our findings may be confirmed. Moreover, studies with a methodology similar to that of the present research conducted in the stages of deciduous and permanent dentition, as well as researches with a longitudinal design can contribute to understanding related to the dimensional changes in the hard palate, which occur throughout the entire duration of occlusal development.

## CONCLUSION

Reference values of normality for the dimensions of the hard palate were suggested, excluding those who presented one or more characteristics that also demonstrated an effect on the hard palate dimensions in the adjusted linear regression analysis, and it was verified that:

1. these dimensions were larger in children of the male sex;
2. in the first transitional period of mixed dentition, the width and depth of the hard palate were smaller than those in the other periods;
3. the width of the hard palate of white children was smaller at the level of the premolars or deciduous molars than it was in non-white children.

Notwithstanding, was verified the reduction in the width of the hard palate in cases of posterior crossbite, Angle’s Class II and III malocclusions, presence of habits on non-nutritional sucking, and in the oronasal respiratory mode.
